# Protocol to study direct (de)phosphorylation events on a proteome-wide scale using on-bead *in vitro* enzyme assays

**DOI:** 10.1016/j.xpro.2026.104629

**Published:** 2026-06-16

**Authors:** Melanie Brunner, Zehan Hu, Marco Caligaris, Michael Stumpe, Claudio De Virgilio, Jörn Dengjel

**Affiliations:** 1 Department of Biology, University of Fribourg, Chemin du Musée 10, 1700 Fribourg, Switzerland

**Keywords:** Cell Biology, Molecular Biology, Protein Biochemistry, Proteomics, Systems biology

## Abstract

Protein phosphorylation regulates essential cellular processes, yet probing kinase and phosphatase activities in native contexts remains challenging. Here, we present a protocol to study direct (de)phosphorylation events on a proteome-wide scale using on-bead *in vitro* kinase and phosphatase assays (OBIKA/OBIPhA). We describe steps for employing active enzyme complexes and identifying direct substrates and regulated phosphosites using mass spectrometry. Compared to traditional *in vitro* approaches, this protocol operates under native conditions to preserve protein interactions and modifications in eukaryotic cell systems.

For complete details on the use and execution of this protocol, please refer to Brunner et al.^1^ and Hu et al.^2^

## Highlights

• Steps for covalently immobilizing native whole-cell extracts onto NHS-activated beads
• Instructions for proteome-wide *in vitro*-kinase and *in vitro*-phosphatase assays
• Guidance for the use of yeast- and mammalian cell-based assays
• Instructions for mass spectrometry sample preparation and phosphopeptide enrichment

## Abstract (editor highlights)

Publisher’s note: Undertaking any experimental protocol requires adherence to local institutional guidelines for laboratory safety and ethics.

## Graphical abstract

**Figure:**
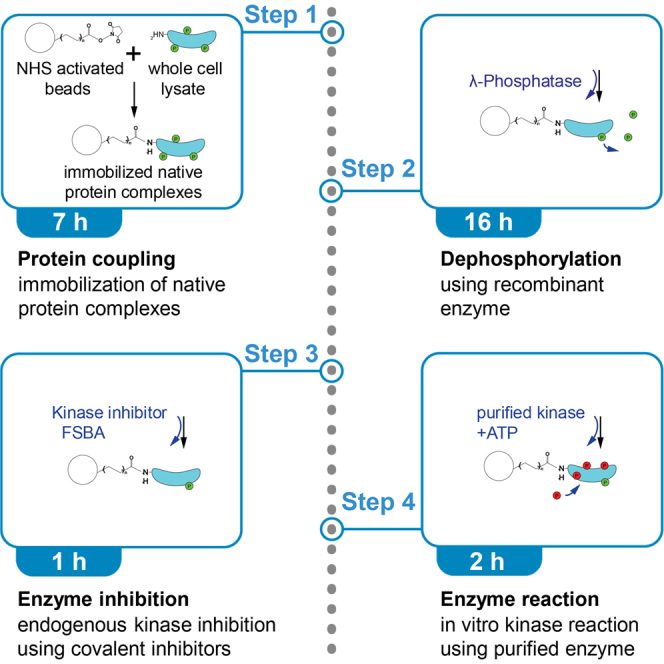

## Before you begin

Cellular signal transduction relies on a sophisticated interplay between protein–protein interactions and post-translational modifications (PTMs), with phosphorylation reactions playing a central role. The phosphate group is unique in carrying two negative charges under physiological pH, and (de)phosphorylation - catalyzed by protein kinases and phosphatases - modulates protein function, localization, stability, and interactions, thereby orchestrating a wide range of cellular responses.^3^ To unravel the complex landscape of protein phosphorylation and identify clinically relevant events, there is a growing demand for high-throughput, sensitive methodologies capable of accurately mapping and quantifying phosphorylation dynamics. Mass spectrometry (MS)-based phosphoproteomics has become the leading approach for global phosphoproteome profiling.^4^

In this protocol, we present a comprehensive phosphoproteomic workflow that combines *in vitro* kinase and phosphatase assays with liquid chromatography-tandem mass spectrometry (LC-MS/MS). Our approach captures the dynamic landscape of the proteome by extracting it under native conditions, thereby preserving quaternary protein structures and protein–protein interactions. Following coupling of the proteome to N-hydroxysuccinimide (NHS)-activated Sepharose beads, which bind to primary amines, i.e., the N-terminal amino group of proteins and the ε-amino group of lysine side chains, *in vitro* phosphorylation or dephosphorylation is performed using purified kinase or phosphatase complexes, respectively. Crucially, covalent inhibitors are used to block endogenous kinase and phosphatase activities, enabling controlled modulation of site-specific (de)phosphorylation.

### Experimental design considerations

Define the biological system and enzyme of interest.

1. Select the cell line, tissue, or organism from which the proteome will be extracted.
2. Ensure availability of purified kinase or phosphatase complexes suitable for *in vitro* reactions.

#### Plan appropriate controls

3. Include reactions without added enzyme to assess background phosphorylation or dephosphorylation.
4. Consider including catalytically inactive enzyme variants or pharmacological inhibitors, if available, to control for non-enzymatic effects.

#### Determine sample scale and replication

5. Estimate the amount of starting material required to achieve sufficient proteome coverage by MS.
6. Plan biological replicates to ensure statistical robustness.

#### Preparation of reagents and equipment

Prepare for native proteome extraction.

7. Ensure that all buffers and equipment required for native lysis are available and (if necessary) pre-cooled.
8. Select covalent inhibitors to irreversibly block endogenous kinases (such as FSBA) or phosphatases (MCLR for serine/threonine phosphatases) prior to *in vitro* reactions.
9. Verify that inhibitors are compatible with downstream enzymatic reactions and MS analysis.

#### Preparation for downstream MS analyses

10. Ensure access to bottom-up phosphoproteomic infrastructure.
11. Confirm availability of enzymatic digestion, desalting, and phosphopeptide enrichment workflows.
12. Arrange access to LC-MS/MS instrumentation.
13. Identify software tools and pipelines for phosphosite identification and quantification.

Completion of these preparatory steps will ensure that the protocol can be executed efficiently, and reproducibly once experimental work begins.

### Innovation

Historically, *in vitro* (de)phosphorylation reactions were coupled to radioisotope labeling using [γ-^32^P]-ATP, with substrate phosphorylation quantified by measuring incorporated radioactivity,^5^ or by using phosphosite-specific antibodies in western blots.^6^ Such assays are highly sensitive and accurate, but typically offer only low to medium throughput. In contrast, MS-based readouts provide a high degree of multiplexing, enabling the simultaneous analysis of thousands of phosphosites.^4^ As a result, MS-based approaches have become the method of choice for studying (de)phosphorylation reactions.

*In vitro* reactions performed at the peptide level or using denatured protein mixtures have been successfully coupled to MS-based readouts to investigate kinase^7^^,^^8^ and phosphatase^9^^,^^10^ target recognition. These approaches are robust, as endogenous enzyme activities are blocked. Peptide arrays or libraries have proven effective for studying primary amino acid sequences, such as kinase and phosphatase motifs.^9^^,^^11^^,^^12^ Additionally, fluorometric multiplexed high-throughput assays based on phosphopeptide libraries have been developed.^13^ However, because secondary and higher-order structures are lost in peptide-based or denatured lysate approaches, it remains unclear whether the identified sites are accessible or functionally relevant *in vivo.*

Our protocol is based on the use of native conditions to preserve quaternary protein structures, thereby minimizing non-specific *in vitro* off-target reactions. As endogenous enzymes remain active, they must be inhibited prior to the addition of purified enzymes of interest. To ensure that these endogenous enzymes remain inactive throughout the workflow, we employ covalent inhibitors, specifically, 5′-fluorosulfonylbenzoyl-5′-adenosine (FSBA) as a protein kinase inhibitor,^8^^,^^14^ and microcystin-LR (MCLR) as a phosphoprotein phosphatase inhibitor.^15^ By integrating *in vitro* and *in vivo* datasets, our protocol allows for the identification of direct, *bona fide* phosphorylation events at the proteome scale - distinguishing primary from secondary modifications (i.e., direct from indirect/downstream modifications by other enzymes), which is not feasible through *in vivo* experiments alone. Subsequent sample preparation includes enzymatic digestion, desalting, and enrichment of low-abundance phosphopeptides prior to LC-MS/MS analysis. The protocol was optimized using mammalian cell lines and purified kinase^2^ or phosphatase^1^ complexes. However, we have also successfully applied this approach to *Saccharomyces cerevisiae*^16^ and *Caenorhabditis elegans*^17^ proteomes, demonstrating its broad applicability across eukaryotic systems.

## Key resources table

**Table:** 

REAGENT or RESOURCE	SOURCE	IDENTIFIER
**Chemicals, peptides, and recombinant proteins**		

Dulbecco’s Modified Eagle Medium (DMEM)	PAN Biotech	Cat#P04-04510
Fetal Bovine Serum (FBS)	BioWest	Cat#S181B-500
YPD Broth Enhanced Formulation w/Peptone Y (Yeast Extract Peptone Dextrose)	US Biological	Cat# Y2075
Phenylmethanesulfonyl Fluoride (PMSF)	Calbiochem	Cat# 52332
Lambda phosphatase	Purified in house	
Trypsin for cell culture	PAN Biotech	Cat#P10-023100
Penicillin-Streptomycin	PAN Biotech	Cat#P06-07100
PP2A inhibitor LB100	RayBiotech	Cat#332-11915-2
PP2A inhibitor okadaic acid	Merck	Cat#O9381
Dulbecco’s Phosphate Buffered Saline (DPBS)	PAN Biotech	Cat#P04-36500
Microcystin LR	Enzo Life Sciences	Cat#ALX-350-012-M001
PhosSTOP	Roche	Cat#04-906-837-001
Proteases Inhibitor Cocktail	Roche	Cat#11-697-498-001
HEPES	Roth	Cat#9105.4
TRIS	Roth	Cat#5429.3
NP-40 Alternative	Millipore	Cat#492016
Sodium chloride (NaCl)	Roth	Cat#3957.4
Manganese (II)-chloride tetrahydrate	Merck	Cat# 1.05927.0100
Magnesium chloride	Roth	Cat# KK36.2
NHS-activated Sepharose 4 fast flow	GE Healthcare	Cat#17-0906-01
Trifluoroacetic acid (TFA)	Sigma-Aldrich	Cat#302031
Formic acid (FA)	Merck	Cat#5.43804.0250
Ammonia solution 25%	Merck	Cat#5438300250
Trypsin	Promega	Cat#V5113
Lys-C	FUJIFILM Wako Pure Chemical Corporation	Cat#129-02541
MS-grade Water	VWR	Cat#23595.328
MS-grade Acetonitrile	VWR	Cat#20060.32
Methanol	VWR	Cat#85855.320
Ethanol	VWR	Cat#153386f
Hydrochloric acid	VWR	Cat#30721-1 L-GL
5’-(4-Fluorosulfonylbenzoyl) adenosine hydrochloride	Sigma Aldrich	Cat#F9128
ATP	Sigma-Aldrich	Cat#A6419
Dithiothreitol	VWR	Cat#0281
Iodoacetamide	Sigma	Cat#4386
Urea	Sigma Aldrich	Cat# U5378
Trypsin	Promega	Cat# V5113
Staurosporine	LC laboratories	Cat# S-9300
Ethylenediaminetetraacetic acid (EDTA)	Sigma Aldrich	Cat# EDS
Ethylene glycol bis(2-aminoethyl ether)-N,N,N′,N′-tetraacetic acid (EGTA)	NeoFroxx	Cat#1752GR025
Active purified kinase complex	NA	NA
Tested kinase: GSK3β	Sino Biological	Cat#10044-H07B
Active purified phosphatase complex	NA	NA
Water with 0.1 % Formic Acid (v/v), Optima LC/MS grade	Fisher Scientific	Cat# LS118
Acetonitrile with 0.1 % Formic Acid (v/v), Optima LC/MS grade	Fisher Scientific	Cat# LS120

**Critical commercial assays**		

BCA protein assay kit	Pierce	cat.no. PIER23225

**Experimental models: Cell lines**		

A549 cells	ATCC	Cat#CCL-185
HeLa cells	ATCC	Cat#CCL-2

**Experimental models: Organisms/strains**		

Yeast strain BY4741	Euroscarf	Cat#Y00000

**Software and algorithms**		

Spectronaut	Biognosys	
R	https://www.r-project.org/	N/A

**Other**		

HR-X columns	Macherey-Nagel	Cat#730936P45
U bottom 96 well plate	Greiner	Cat# 650201_100
Sapphire PCR microplates 96 well	Greiner	Cat# 652270
Bravo automated liquid handling platform	Agilent	
Fe (III)-NTA cartridges (5 μL)	Agilent	Cat# G5496-60085
Dialysis Tubing Cellulose Membrane (MWCO: 14,000)	Sigma-Aldrich	Cat #D0405
Lyophilizer		
Orbitrap Astral mass spectrometer	Thermo Fisher Scientific	
Vanquish Neo UHPLC system	Thermo Fisher Scientific	
Dialysis Tubing (molecularporous membrane tubing, MWCO: 3,500)	SPECTRUM	Cat# 1977.1
FastPrep-24™ 5G bead beating grinder and lysis system	MP Biomedicals	
Filtration unit made of 90 mm Glass Microanalysis Holder	Sterlitech Corporation	
Acid-washed glass beads	Retsch	Cat# 22.222.0002
NanoDrop One Microvolume UV-Vis Spectrophotometer	Thermo Scientific	Cat#ND-ONE-W

## Materials and equipment

**Table:** Lysis buffer mammalian cells

*Reagent*	Final concentration
HEPES pH 8.0	50 mM
NP-40	1%
NaCl	150 mM
Protease inhibitor cocktail	1x

Storage at 20**–**25°C for up to one month.

**CRITICAL:** Add protease inhibitor freshly to the buffer!

**Table:** Lysis buffer yeast cells

Reagent	Final concentration
HEPES pH 7.5	50 mM
NP-40	0.1%
NaCl	150 mM
EDTA pH 8.0	1 mM
EGTA pH 8.0	1 mM
Protease inhibitor cocktail	1x

Storage at 20**–**25°C for up to one month.

**CRITICAL:** Add protease inhibitor freshly to the buffer!

**Table:** Dialysis buffer yeast cells

Reagent	Final concentration
HEPES pH 7.5	50 mM
NP-40	0.1%
NaCl	150 mM
EDTA pH 8.0	1 mM
EGTA pH 8.0	1 mM
PMSF	1 mM

Storage at 20**–**25°C for up to one month.

**Table:** Beads washing buffer

Reagent	Final concentration
HEPES pH 8.0	50 mM
NP-40	0.1%
NaCl	150 mM

Storage at 20**–**25°C for up to one month.

**Table:** 10x phosphatase buffer

Reagent	Final concentration
HEPES pH 8.0	500 mM
NP-40	1%
NaCl	1 M

Storage at 20**–**25°C for up to one month.

**Table:** 10x kinase buffer

Reagent	Final concentration
Tris-HCl pH 7.6	500 mM
MgCl_2_	10 mM
NaCl	1.5 M

Storage at 20**–**25°C for up to one month.

**Table:** 10x kinase inhibition buffer

Reagent	Final concentration
HEPES pH 7.6	500 mM
MgCl_2_	10 mM
NaCl	1.5 M

Storage at 20**–**25°C for up to one month.

**Table:** Urea buffer

Reagent	Final concentration
Urea	8 M
Tris HCl pH 8	50 mM

Prepare fresh before usage.

**Table:** Dithiothreitol (DTT) stock solution

Reagent	Final concentration
DTT	100 mM
ddH_2_O	-

Storage at −20°C for up to three months.

**Table:** Iodoacetamide (IAA) stock solution

Reagent	Final concentration
IAA	550 mM
ddH_2_O	**–**

Storage at −20°C in dark tubes for up to three months.

## Step-by-step method details

### Preparation of lysate

**Timing: 1 h(for mammalian cells)**

**Timing: 6 h (up to 14 h = overnight)(for yeast cells)**

In this step, cells are lysed to generate native whole-cell protein extracts, and the lysates are standardized to a defined protein concentration. For yeast cells, dialysis is applied.

1. For mammalian cell lines
  a. Grow cells in DMEM to near confluence (∼80%) in 15-cm dishes
  b. Place cells on ice. Aspirate medium and rinse twice with 15 mL ice cold PBS
  c. Collect the cells using a cell scraper, transfer them into 50-mL falcon tubes and pellet them by centrifugation (2000 *g* for 3 min at 4°C). Discard supernatant.
    **Pause point:** Cell pellets can be stored at −80°C
  d. Add lysis buffer to the cell pellets at 4°C; the ratio of sample/lysate buffer should be at least 1:3 (vol/vol).
  e. Homogenize the lysate by vortexing every 5 min.
  f. After 20 min, pellet cell debris by centrifugation (3000 *g* for 10 min at 4°C) and keep the supernatants.
  g. For OBIKA only (for OBIPhA directly jump to: Phosphorylation): determine protein concentration by BCA protein assay. The lysate concentration should be ∼10 mg/mL.
2. For yeast cells.
  a. Pre-grow cells overnight in 70 mL YPD at 30°C.
  b. The day after, dilute the cells at 0.2 OD_600nm_/mL in 2 L YPD.
  c. Grow cells at 30°C until 2 OD_600nm_/mL. If needed, treat with drugs to inhibit the kinase of interest.
  d. Collect cells with filtration unit (max 1 L for each filter)
  e. Collect the cells from the filter with a spatula and transfer them into a 5 mL syringe. Then, rapidly freeze the cells by dispensing them from the syringe directly above a small tank of liquid nitrogen.
  f. Transfer the frozen cell pellets into a 50 mL falcon tube.
    **Pause point:** Cell pellets can be stored at −80°C
  g. Split cell pellets in 2 new 50 mL falcon tubes.
  h. Cells are lysed in 10 mL lysis buffer (5 mL per tube) in the presence of acid-washed glass beads using a FastPrep-24™ homogenizer (6x30 s with 60 s pause after each cycle for 2 cycles at 4°C).
  i. Separate the cell lysate from the acid-washed glass beads by puncturing the tube with a needle and expelling the lysate through the holes using a 50 mL syringe. Then, transfer the collected lysate to a new 50 mL falcon tube.
  j. Clarify the lysate by centrifugation at the highest speed for 5 min at 4°C.
  k. Transfer the clear cell lysate to a new 15 mL tube.
  l. Protein concentration can be measured by BCA, samples should be diluted to 10 mg/mL.
3. Dialysis
  This step is specific for yeast lysates only.
  a. 2 x 12 cm dialysis tubing (1 mL/1 cm) is prewashed with dialysis buffer.
  b. Seal one side of the tubing with tubing closure.
  c. Fill the tubing with dialysis buffer and check whether it is leaking. If not, remove the buffer and fill the tubing with lysate.
  d. Seal the other side with closure. Check again for leaking.
  e. Place the tubing in 2 L beaker. The beaker is filled with 2 liters of dialysis buffer.
  f. Put a magnetic stir bar into the beaker and keep it on a Magnetic stirrer at 500 rpm in 4°C/cold room.
  g. After 2 h change the buffer and incubate at 500 rpm, 4°C for 4 h or overnight.
  h. Next day, transfer the dialyzed lysate into two 15 mL falcon tubes and remove precipitates by centrifugation at the highest speed for 5 min at 4°C. Supernatant is kept on ice. Protein concentration/amount can be measured by BCA assay.

### OBIPhA only: Phosphorylation

**Timing: 2 h**

In this step, endogenous phosphatase activity is inhibited, and ATP is supplied to promote maximal protein phosphorylation by endogenous kinases in the lysate.

4. Add 1 μM MCLR and 1 mM ATP for 2 h at 37°C, shaking
5. Perform protein assay (BCA) to determine protein concentration. The lysate concentration should be ∼10 mg/mL.

### NHS bead coupling

**Timing: 7 h or overnight**

In this step, the total proteome is covalently immobilized onto NHS-activated beads, enabling efficient buffer exchange and downstream handling.

6. Take 0.5 x n mL beads (bed volume, n = number of samples) and wash 3 times with 10 mL ice cold 1 mM HCl. This step can be done on an Econo-Column. Keep upper bead level straight.

**CRITICAL:** Beads are big, cut pipette tips to ensure proper pipetting! Based on binding test, 1 mL beads (bed volume) bind to 10 mg protein, here we use less beads and more protein to saturate the beads (1 mL of lysate per sample).

7. Wash the beads twice with 10 mL beads washing buffer.
8. Mix the beads with lysate in a 50 mL falcon tube and incubate the mixture on the rotor at 4°C for 4**–**6 h at 20**–**25°C or overnight in the cold room.

**CRITICAL:** Until this step, the buffer should not contain any compounds which have primary amines.

9. Spin down the beads at 2000 *g* for 5 min at 4°C and remove the supernatant.
10. Measure the protein concentration of supernatant using the BCA protein assay to evaluate the binding efficiency.

### OBIKA only: Dephosphorylation

**Timing: Overnight (12–16 h)**

In this step, the proteome is being dephosphorylated to decrease the level of endogenous protein phosphorylation as much as possible for the maximal identification of subsequent *in vitro* kinase reactions.

11. Wash the beads with 10 mL of 1 x phosphatase buffer, spin down at 2000 *g* for 5 min at 4°C and remove the supernatant.
12. Repeat 3 times and transfer into a 15 mL falcon tube.
13. Resuspend the beads in 1 x phosphatase buffer with 1 x MnCl_2_ solution up to 6 mL.
14. Add 5,000 to 10,000 units of Lambda phosphatase.
15. Incubate on a rotor/shaker at 30°C for 1 h and then 4°C overnight in order to dephosphorylate the proteome completely.

### Kinase assay: OBIKA

**Timing: 6 h**

In this step, endogenous kinases are inhibited covalently first. Subsequently the kinase-of-interest or a control is added to phosphorylate its substrates.

16. Spin down beads at 2000 *g* for 5 min, 4°C and remove the supernatant.
17. Wash beads 2 times with 10 mL of 1 x kinase inhibition buffer, spin at 2000 *g* for 5 min, 4°C each time
18. Add 20 μl of FSBA solution per mL (final concentration: 1 mM) to inhibit endogenous kinases.
19. Incubate the beads on rotor/wheel for 1 h at 20**–**25°C.
20. Wash the beads 3 times with 10 mL of 1 x kinase buffer to remove inhibitors, spin down at 2000 *g* for 5 min, 4°C, remove supernatant.
21. During the last washing step, split the beads into nine 2 mL low binding Eppendorf tubes (3 tubes per condition: (I) neg. ctrl with ATP/without kinase; (II) kinase samples (with ATP/with kinase), (III) kinase dead/inhibited kinase samples (with ATP/with kinase)).

**CRITICAL:** The control is dependent on your sample setup and can be ATP-only (without kinase), mutated kinase or kinase & inhibitor (check that inhibitor is applicable for *in vitro* use!)

22. Spin down beads and remove supernatant.
23. Remove the remaining buffer with gel loading tips and fill the tubes with kinase buffer with 1 x PhosSTOP up to 1350 μL.
24. Add 150 μl of ATP solution and n μL of active kinase or ctrl buffer. Incubate the kinase reaction tube 2**–**4 h on thermomixer at 37°C for mammalian kinase, 30°C for yeast kinase.

**CRITICAL:** The incubation time is dependent on the activity and amount of kinase. Activity should be checked beforehand.

25. Spin down the beads and remove the supernatant.
26. Wash the beads three times with 1 mL of kinase buffer.

**CRITICAL:** Steps 25 and 26 are recommended if detergent is present in the purified kinase complex which negatively affects MS identification rates due to ion interferences.

27. Freeze the beads in liquid nitrogen and dry the beads in lyophilizer overnight.

### Phosphatase assay: OBIPhA

**Timing: 4–6 h**

In this step, the phosphatase-of-interest is dephosphorylating its substrates and negative controls do not.

28. Spin down beads at 2000 *g*, for 5 min at 4°C and remove supernatant.
29. Resuspend beads with 1 x phosphatase buffer with 1 x MnCl_2_ stock up to n mL (n = number of samples).
30. Split beads into low binding 2 mL Eppendorf tubes.
31. Add phosphatase elution buffer to the negative Ctrl samples, add lambda-phosphatase to the positive Ctrl samples, add the phosphatase with its respective inhibitor to the inhibited samples, and add purified active phosphatases to the experiment samples.
32. Incubate the phosphatase reaction tubes 2**–**4 h at 37°C for mammalian phosphatases, 30°C for yeast phosphatases, with shaking.

**CRITICAL:** The incubation time is dependent on the activity and concentration of phosphatases. Activity should be checked beforehand.

33. Freeze the beads in liquid nitrogen and dry the beads in lyophilizer overnight.

### Digestion

**Timing: 2 h plus a 14 h/overnight digestion period**

In this step, disulfide bonds are first reduced, followed by alkylation. Subsequently, proteins are enzymatically digested to generate peptides suitable for downstream MS–based phosphoproteomic analysis.

34. Add 250 μL of urea buffer with 1 mM DTT to the dry beads, incubate for 30 min at 1000 rpm on a shaker at 20**–**25°C.

**CRITICAL:** DTT is oxygen sensitive and should therefore be prepared freshly before use. Beads will expand compared to dried state, therefore 250 μL might not be sufficient to solvate all beads. If so, you either switch to a bigger tube and add more urea buffer, or you dilute your samples to 4 M urea which is sufficient for Lys-C digestion.

35. Add 2.25 mM IAA, incubate at 20**–**25°C in the dark at 1000 rpm, for 30 min.

**CRITICAL:** IAA is light sensitive; incubations should therefore be performed in the dark.

36. Add 25 μg Lys-C (1:100 of expected protein amount) to each tube and incubate at 20**–**25°C for 2**–**4 h on the rotor/wheel.
37. Dilute with Tris buffer to 1 M urea (2 mL)

**CRITICAL:** For trypsin to work, urea concentration should be equal to or less than 1 M

38. Add 25 μg trypsin (1:100 p/p) and incubate at 20**–**25°C overnight on the rotor/wheel.

### SPE peptide purification

**Timing: 4 h**

In this step, peptides are purified using solid-phase extraction (SPE) to remove salts and other contaminants prior to phosphopeptide enrichment and MS analysis.

**CRITICAL:** Acetonitrile is a flammability hazard category 2, toxic hazard category 4 and eye irritant category 2. Wear proper personal protective equipment (PPE) when handling and avoid contact with your eyes. Keep away from heat, sparks and open flame. Use per safety data sheet (SDS) recommendations.

39. Add 50% TFA to reduce pH of samples to ∼pH 2.5.
40. Purify the peptides using HR-X reversed phase cartridges.
41. Prime the cartridges with 2 Vol (4 mL) of MeOH and 2 Vol (4 mL) of Buffer B L-pH.
42. Equilibrate the cartridges with 3 Vol (6 mL) Buffer A L-pH.
43. Load sample (including the beads) on HR-X cartridges.
44. Wash the columns with 10 mL of Buffer A L-pH.
45. Elute the peptide with 6 mL of Buffer B L-pH.
46. Freeze the samples in liquid nitrogen and dry samples in lyophilizer overnight.

**Pause point:** Samples can be stored at −80 °C for up to 6 months.

### Phosphopeptide enrichment

**Timing: 3 h**

In this step, phosphorylated peptides are selectively enriched from the total peptide mixture using IMAC (FeNTA) to improve the phosphoproteome coverage.

47. Resuspend the peptides in 200 μl equilibration buffer and load samples on the 96-well sample plate on the Bravo automated liquid handling platform
48. Run the phosphopeptide enrichment application.
49. Dry the phospho-samples & non phosphorylated flowthrough in lyophilizer and resuspend in 20 μL of 0.1% formic acid for LC-MS/MS analysis.
50. Measure the peptide concentration using a NanoDrop spectrophotometer and adjust to 100 ng/μL.

**Pause point:** Samples can be stored at −80 °C for up to 3 months.

### MS measurements and data analysis

In this step, the peptides or phosphopeptides are analyzed by high-resolution LC-MS/MS to identify and quantify phosphorylation sites. This is followed by data processing for peptide identification, site localization, quantification and statistical evaluation of events. 1 h per sample.

51. Introduce 250 ng per sample into the autosampler of a nano-HPLC instrument and operate LC-MS/MS system in DIA mode.
52. Transfer .raw files to the data analysis computer and load them into DIA-NN or Spectronaut respectively, for processing.
53. Use the generated files of your analysis software to perform data analysis and evaluation of performance. This can be done with any statistical software.

## Expected outcomes

**Figure 1:**
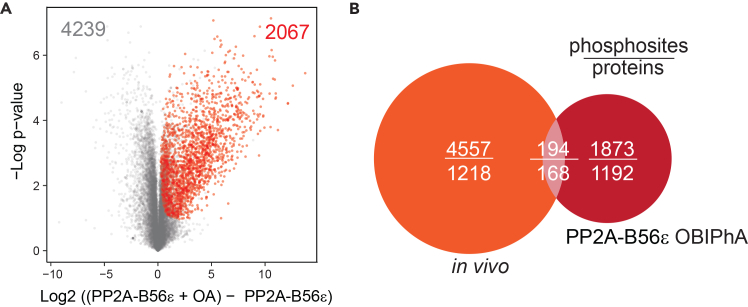
OBIPhA results (A) Volcano plot highlighting significantly regulated phosphosites for PP2A-B56ε complexes being OA sensitive (n = 3 biological replicates). Significant sites are highlighted in red (FDR <0.05). Numbers of quantified and regulated sites are indicated in grey and red font respectively. (B) Comparison between *in vivo* and OBIPhA results. *Bona fide* PP2A-B56ε target sites should be regulated both *in vivo* and *in vitro*, i.e., 194 sites on 168 proteins. Data was adapted from Brunner et al.^1^ DIA data was analyzed using Spectronaut version 19.

**Figure 2:**
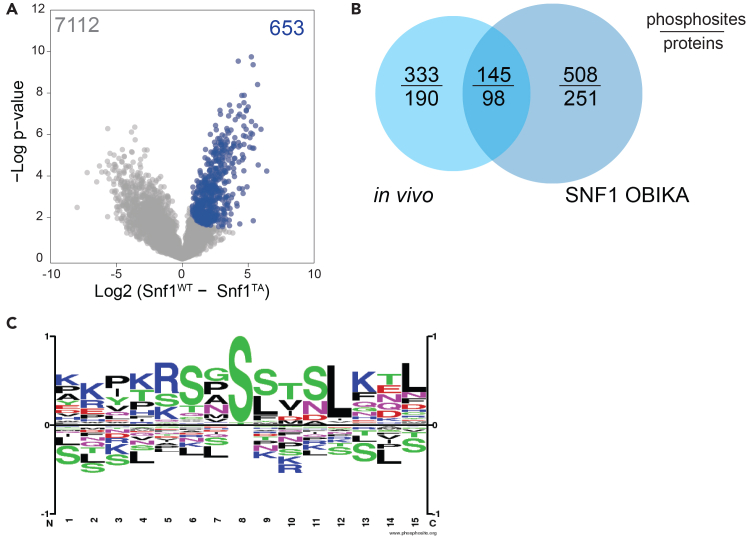
OBIKA results (A) Volcano plot highlighting significantly regulated phosphosites for SNF1 complexes (n = 5 biological replicates). Immobilized proteome was incubated with purified SNF1 complexes containing either wild-type Snf1 (Snf1^WT^) or with the kinase-inactive Snf1^T210A^ mutant (Snf1^TA^). Significant sites are highlighted in blue (FDR <0.05). Numbers of quantified and regulated sites are indicated in grey and blue font respectively. (B) Comparison between *in vivo* and OBIKA results. *Bona fide* SNF1 target sites should be regulated *in vivo* and *in vitro*, i.e., 145 sites on 98 proteins. (C) Kinase motif analyses of Snf1 phosphosites identified by *in vivo* and *in vitro*. Data was adapted from Caligaris et al.^16^

The number of identified and quantified phosphosites depends on the mass spectrometer used. With the latest generation of instruments, we anticipate that data-independent acquisition (DIA) can quantify at least 10,000 phosphorylation sites per run. Using a minimum of three biological replicates and comparing wild-type enzymes with either pharmacologically inhibited enzymes (Figure 1A) or catalytically inactive variants (Figure 2A), several thousand *in vitro*-regulated phosphorylation sites can be identified.

To filter for physiologically relevant, *bona fide* target sites, we routinely compare *in vitro* results with corresponding *in vivo*, i.e., *in cellulo*, phosphoproteomic datasets, typically yielding more than 100 validated target sites (Figures 1B and 2B). In the case of protein kinases, these target sites can be used to derive kinase motifs, providing insights into substrate specificity (Figure 2C). In case of the SNF1 and ULK1 complexes we could nicely corroborate published kinase motifs.^18^^,^^19^^,^^20^^,^^21^^,^^22^^,^^23^

## Quantification and statistical analysis

***Note:*** Raw files can be analyzed using various software (*e.g.* Spectronaut, DIA-NN).

**Figure 3:**
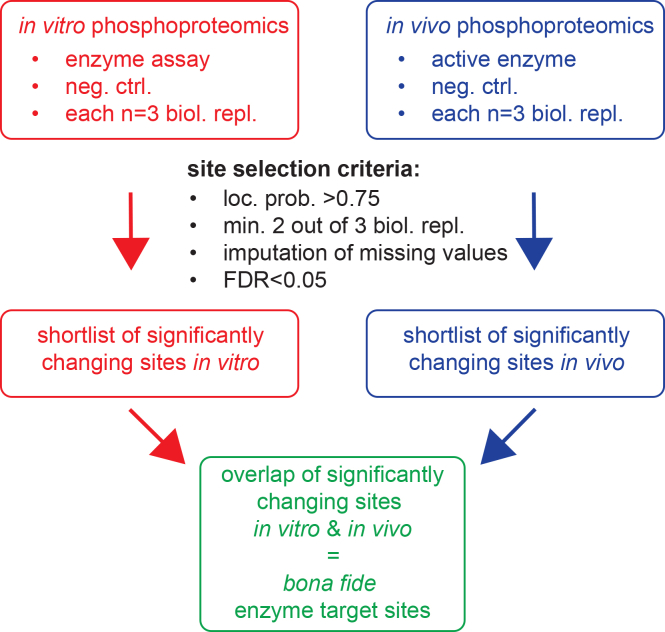
Decision tree for target site identification Shown are the filtering steps to shortlist in vitro and in vivo regulated sites for the identification of direct enzyme target sites.

Data analysis is performed using Perseus version 2.1.6.0. Phosphorylation sites with a maximum localization probability ≥ 0.75 are considered for analysis (see Figure 3 for decision tree). For OBIKA, phosphosites must have minimally two out of three valid values in kinase samples. In these samples, missing values are imputed by condition-wise singular value decomposition (SVD). Missing values in negative control experiments are replaced by random values of a normal distribution to mimic low abundance measurements. Both width and down shift are applied according to default settings. Replicates are grouped and significant changes determined (FDR < 0.05).

For OBIPhA, SVD is used for imputing missing values in control and inhibitor samples that are quantified in at least two replicates. Missing values in phosphatase measurements are replaced by random values of a normal distribution to mimic low abundance measurements. Both width and down shift are applied according to default settings. Replacement of missing values is applied to each expression column separately. Replicates are grouped and significant changes determined (FDR < 0.05).

For *in vivo* data, phosphorylation sites with a maximum localization probability ≥ 0.75 are considered for analysis (see Figure 3 for decision tree). Each site was normalized to the respective protein intensity. Treated samples need at least two out of three valid values per condition, control samples were imputed using random values of a normal distribution to mimic low abundance measurements. Replicates were grouped, significant changes were determined using FDR ≤ 0.05 as a threshold. To define direct substrates, significant phosphosites (FDR ≤ 0.05) from OBIKA/OBIPhA *in vitro* experiments were intersected with significant hits from the corresponding *in vivo* dataset. Overlap was determined by exact site matching (Uniprot ID + residue site). Sites reproducibly regulated across both experimental contexts were classified as direct, bona fide substrates.

Using a confusion matrix to compare phosphosites detected and quantified by both *in vitro* and *in vivo* experiments one can determine true and false, positives and negatives. Whereas true positives and negatives can be readily defined as the sites significantly changing and being stable in both settings, respectively, and false positives as sites changing significantly only in one of them, the determination of false negatives is not trivial as both *in vitro* and *in vivo* experiments do not exclusively yield “true”, i.e., direct, enzyme target sites.

## Limitations

The limitations of this protocol reflect general challenges associated with *in vitro* assays and unbiased proteomics. Although we perform reactions under native conditions to preserve complex protein structures, off-target effects cannot be entirely ruled out due to the artificial nature of *in vitro* setups. The loss of cellular compartmentalization during cell lysis may result in phosphorylation of non-physiological targets. Additionally, the disruption of membrane structures can lead to partial unfolding of substrates, potentially exposing phosphorylation sites that would be sterically inaccessible under *in vivo* conditions. To mitigate these limitations, we always complement our *in vitro* assays with *in vivo* phosphoproteomics and focus only on phosphorylation events identified by both approaches.^1^^,^^2^^,^^16^

Another limitation lies in the detection and quantification capabilities of MS, which are inherently abundance-dependent. Phosphorylated peptides from highly abundant proteins are more likely to be detected than those from low-abundance proteins. Moreover, the dynamic range of modern mass spectrometers typically spans three to six orders of magnitude.^24^ As a result, highly abundant peptides may mask lower-abundance ones, even if the instrument is sensitive enough to detect them in principle. Complexity of low abundance sites can lead to misassignment of sites due to missing discriminative ions. In addition, substrates falling consistently below the detection threshold may be absent from the final substrate list, introducing a bias toward high-abundance targets.

Finally, defining direct substrates by overlap between *in vitro* and *in vivo* datasets reduces but does not eliminate false discoveries. Requiring significance in both experiments lowers the probability of false hits, but this conservative approach increases the false negative rate. True substrates failing to reach significance in one dataset due to biological variability, differences in cellular context, or technical limitations will be excluded from the final substrate repertoire. These considerations should be kept in mind when interpreting the completeness of the identified substrate list.

## Troubleshooting

### Problem 1

The protein yield is lower than expected/necessary (Preparation of lysate).

### Potential solution

• Might be due to incomplete cell lysis or inadequate buffer volume. Increase lysis buffer:pellet ratio to 3:1 (v/v), extend vortexing, and verify protease inhibitor activity

### Problem 2

Poor protein binding (NHS Bead coupling).

### Potential solution

• Beads not properly activated or lysate contains amines; Beads might be too old. Wash beads thoroughly with 1 mM HCl; ensure that no Tris or primary amine-containing buffers are used before coupling

### Problem 3

Incomplete removal of phosphorylation sites prior to kinase assays (OBIKA only: Dephosphorylation).

### Potential solution

• Insufficient λ-phosphatase activity: Verify phosphatase activity using a test substrate; extend incubation overnight at 30°C

### Problem 4

Weak phosphorylation signal (Kinase assay - OBIKA).

### Potential solution

• Endogenous kinases not completely inhibited before the reaction, leading to high background phosphorylation, Verify full inhibition of endogenous kinases with FSBA before adding the active kinase; add 2 μL staurosporine solution per mL (Final conc. 0.1 mM) as an additional non-covalent kinase inhibitor and incubate beads on the rotor/wheel for 30 min at 20**–**25°C. Due to the higher affinity of staurosporine, FSBA should be added prior to staurosporine to inhibit the kinases covalently.
• Purified kinase is inactive. Confirm activity of purified kinase on a test substrate (e.g., with site specific western blot) e.g., GSK3β (Sino Biological, art. Nr.10044-H07B) was tested for Myc pT58 phosphorylation signal by western blot

### Problem 5

Peptide recovery low or incomplete digestion (Digestion).

### Potential solution

• Urea concentration too high or insufficient amount of enzyme is used: Dilute to ≤1 M urea before adding trypsin; ensure min. enzyme:substrate ratio 1:100

### Problem 6

Peptide loss (SPE peptide purification).

### Potential solution

• Cartridge not properly equilibrated: Precondition columns with methanol, buffer B and buffer A, avoid drying of cartridge.

### Problem 7

Low phosphopeptide yield (Phosphopeptide enrichment).

### Potential solution

• Fe(III)-NTA cartridges overloaded or washing too stringent: Reduce peptide load per cartridge; use recommended flow rate (5 μL/min)

### Problem 8

Variable phosphosite identification (MS measurements and data analysis).

### Potential solution

• Mismatching of DIA data: Generate a phosphopeptide library and search data against library

### Problem 9

Variable phosphosite quantification (MS measurements and data analysis).

### Potential solution

• Inconsistent loading or poor spray stability: Check autosampler precision, recalibrate ESI spray, ensure column free of clogs.
• Neg. controls are not completely inhibited: use specific inhibitors of enzymes of interest and test e.g., by site-specific western blot that enzymes are truly inhibited.

## Resource availability

### Lead contact

Further information and requests for resources and reagents should be directed to and will be fulfilled by the lead contact, Jörn Dengjel (joern.dengjel@unifr.ch).

### Technical contact

Technical questions on executing this protocol should be directed to and will be answered by the technical contact, Melanie Brunner (melanie.brunner@unifr.ch).

### Materials availability

Generated materials are freely available via the lead contact.

### Data and code availability

• Discovery proteomics data are freely available via the PRIDE repository.
• This paper does not report original code.
• Any additional information required to reanalyze the data reported in this paper is available from the lead contact upon request.

## Acknowledgments

This work was supported by the University and the Canton of Fribourg as part of the SKINTEGRITY.CH research network (J.D.), the 10.13039/501100001711Swiss National Science Foundation (184671/214824 to C.D.V., 212187 to J.D., and 229588 to C.D.V. and J.D.). We thank Jesper V. Olsen, Luisa M. Schmidt and the rest of his group at the University of Copenhagen for hosting J.D. and supporting the finalization of this manuscript.

## Author contributions

Conceptualization, M.B., Z.H., and J.D.; methodology, M.B., Z.H., M.C., and M.S.; formal analysis, M.B., Z.H., M.C., and M.S.; writing – original draft, M.B., Z.H., M.C., C.D.V., and J.D.; writing – review and editing, M.B., Z.H., M.C., M.S., C.D.V., and J.D.; visualization, M.B., Z.H., and J.D.; funding acquisition, C.D.V. and J.D.

## Declaration of interests

The authors declare no competing interests.
